# Individualisierung der antihypertensiven Therapie bei Diabetes mellitus. Leitlinie der Österreichischen Diabetes Gesellschaft (Update 2026)

**DOI:** 10.1007/s00508-026-02719-7

**Published:** 2026-04-30

**Authors:** Christoph H. Saely, Gerit-Holger Schernthaner, Johanna Maria Brix, Renate Klauser-Braun, Emanuel Zitt, Heinz Drexel, Guntram Schernthaner

**Affiliations:** 1 VIVIT Institut Feldkirch, Feldkirch, Österreich; 2 Private Universität im Fürstentum Liechtenstein, Liechtenstein, Liechtenstein; 3 Abteilung für Innere Medizin I, LKH Feldkirch, Feldkirch, Österreich; 4 Klinische Abteilung für Angiologie, Universitätsklinik für Innere Medizin II, Medizinische Universität Wien, Wien, Österreich; 5 1. Medizinische Abteilung mit Diabetologie, Endokrinologie und Nephrologie, Klinik Landstraße, Wien, Österreich; 6 Karl Landsteiner Institut für Adipositas und Stoffwechselerkrankungen, Wien, Österreich; 7 3. Medizinische Abteilung, Klinik Donaustadt, Wien, Österreich; 8 Innere Medizin III, LKH Feldkirch, Feldkirch, Österreich; 9 Drexel University College of Medicine, Philadelphia, PA USA; 10 Klinische Abteilung für Angiologie, Universitätsklinik für Innere Medizin II, Universität Wien, Wien, Österreich

**Keywords:** Multimodale Therapie, Therapiekombinationen, Risikofaktoren, Outcome, Reduktion von Komplikationen, Multimodal therapy, Combination therapy, Risk factors, Outcome, Reduction of complications

## Abstract

Hypertonie ist eine sehr häufige Komorbidität bei Diabetes mellitus, die – wenn unzureichend behandelt – signifikant zur erhöhten Mortalität und zum Auftreten von mikrovaskulären und makrovaskulären Komplikationen beiträgt. Blutdruckzielwerte um 120–129/70–79 mm Hg waren in den Studien mit der besten Prognose assoziiert, wobei die Blutdruckzielwerte je nach Alter und Komorbiditäten individualisiert werden sollten; besonders wichtig ist, dass ein Blutdruck < 140/90 mm Hg erreicht wird. Für die meisten Menschen mit Diabetes ist eine Kombinationstherapie notwendig, wobei Medikamente mit gut evidenzbasierter Reduktion des kardiovaskulären Risikos (neben ACE-Hemmern und Angiotensinrezeptorblockern Dihydropyridin-Calciumantagonisten und Thiaziddiuretika) eingesetzt werden sollten, präferenziell als Kombinationspräparate. Nach Erreichung der Zielwerte muss die antihypertensive Therapie fortgeführt werden, wobei regelmäßige Selbstmessungen des Blutdrucks für die Optimierung der Blutdruckeinstellung sehr hilfreich sind. SGLT-2-Inhibitoren oder GLP-1-Rezeptoragonisten tragen ebenfalls zur Blutdrucksenkung bei Diabetes bei. Dies gilt auch für den seit der letzten Auflage der Leitlinien neu verfügbaren kombinierten GLP-1/GIP-Rezeptoragonisten Tirzepatid. Seit der letzten Auflage der Leitlinien zeigten Interventionsstudien Vorteile einer intensiveren Blutdrucksenkung bei Typ-2-Diabetes, was sich in den aktuellen Leitlinien internationaler Gesellschaften widerspiegelt und auch in die vorliegenden Empfehlungen übernommen wird; die Individualisierung der Blutdrucktherapie bleibt aber eine Kernempfehlung der Österreichischen Diabetes-Gesellschaft.

## Abstract (en)

Hypertension is one of the most important comorbidities of diabetes, significantly contributing to increased mortality and leading to macrovascular and microvascular complications. When assessing the medical priorities for patients with diabetes, treating hypertension should be a primary consideration. In the present review practical approaches to hypertension in diabetes, including individualized targets for preventing specific complications are discussed according to the current evidence and guidelines. Blood pressure values of 120–129/70–79 mm Hg are associated with the best outcome; most importantly, at least blood pressure values < 140/90 mm Hg should be achieved in most patients. Most patients with diabetes require combination therapy to achieve blood pressure goals; agents with clear evidence of cardiovascular risk reduction should be used (including, besides angiotensin-converting enzyme inhibitors and angiotensin receptor blockers, dihydropyridine calcium channel antagonists and thiazide diuretics), preferable in single pill combinations. Once the target is achieved, antihypertensive drugs should be continued. The use of SGLT‑2 inhibitors and GLP‑1 receptor agonists contribute to blood pressure lowering in diabetes. This also holds true für the newly available combined GLP-1/GIP receptor agonist tirzepatide. Since the last release of these guidelines evidence from clinical trials has become available that supports more stringent blood pressure control in diabetes, which is reflected in international guidelines and has also been adopted in these recommendations. The individualization of blood pressure management, however, remains a core recommendation of the Austrian Diabetes Association.

## Definition

Eine arterielle Hypertonie soll entsprechend den aktuellen Leitlinien der Europäischen Kardiologischen Gesellschaft (ESC) dann diagnostiziert werden, wenn der Blutdruck bei wiederholten Messungen an verschiedenen Tagen ≥ 140/90 mm Hg liegt; Blutdruckwerte von 120–139/70–89 mm Hg werden als erhöhter Blutdruck bezeichnet [1]. Es werden in den USA niedrigere Grenzwerte für die Diagnose einer Hypertonie vorgeschlagen [2, 3]; diese Definition wird allerdings kontrovers diskutiert [4–6], und die vorliegenden österreichischen Leitlinien [7–9] orientieren sich in Bezug auf die Definition von arterieller Hypertonie und erhöhtem Blutdruck an den aktuellen Empfehlungen der ESC.

## Epidemiologie und Screening

Die arterielle Hypertonie ist eine sehr häufige Komorbidität sowohl bei Menschen mit Typ-2- als auch bei Menschen mit Typ-1-Diabetes mellitus, und ein erhöhter Blutdruck ≥ 140 mm Hg systolisch und/oder ≥ 90 mm Hg diastolisch findet sich in Abhängigkeit von Diabetesdauer und Vorliegen bzw. Ausmaß einer Adipositas in 50–60 % aller Betroffenen [10]. Der Blutdruck sollte bei jeder Visite eines Menschen mit Diabetes kontrolliert werden. Hypertensive Menschen mit Diabetes sollten ihren Blutdruck auch zu Hause selbst regelmäßig kontrollieren. Wichtig ist eine korrekte Messtechnik: sitzend, den Arm auf Herzniveau aufgestützt, den Rücken angelehnt, die Beine am Boden, nach 5 min Ruhe mit einer dem Armumfang angepassten Manschettenbreite. Neben ärztlichen Blutdruckmessungen sollen auch häusliche Blutdruckmessungen erfolgen. Die ambulante Langzeitblutdruckmessung gibt wertvolle zusätzliche Informationen zum Blutdruckverlauf im Tagesverlauf und vor allem zur Blutdrucksituation in der Nacht. Häufig geht die Hypertonie dem Diabetes zeitlich voraus und findet sich dabei assoziiert mit anderen Komponenten des Insulinresistenzsyndroms; Menschen mit Hypertonie haben ein deutlich erhöhtes Risiko, einen Diabetes zu entwickeln [11]. Sie sollen deshalb auf das Vorliegen einer Glukosestoffwechselstörung gescreent werden.

Im Vergleich zu Menschen ohne Diabetes findet man insbesondere bei älteren Menschen mit Diabetes häufig eine isolierte systolische Hypertonie. Das häufige Fehlen einer physiologischen Nachtabsenkung („Dipping“) geht mit einem erhöhten Risiko für Albuminurie bzw. Linksventrikelhypertrophie einher und ist ein ungünstigster Prädiktor für kardiovaskuläre Ereignisse [9].

## Lebensstilinterventionen zur Blutdrucksenkung

Lebensstilmaßnahmen sind die Basis jeder antihypertensiven Therapie. Bereits bei Menschen mit Blutdruckwerten > 120/70 mm Hg sollten eine Gewichtsreduktion bei Übergewicht oder Adipositas, eine Kochsalz-arme und Kalium-reiche Diät, der Verzicht auf übermäßigen Alkoholkonsum sowie körperliche Aktivität empfohlen werden. In der Initialphase der LOOK-AHEAD-Studie (*n* = 5145) senkte eine Gewichtsreduktion von ca. 8 kg nach einem Jahr bei übergewichtigen/adipösen Menschen mit Typ-2-Diabetes die Blutdruckwerte um 5/2 mm Hg signifikant [12]; bei einem Gewichtsverlust > 15 % betrug die systolische Blutdrucksenkung sogar 10 mm Hg.

## Individualisierung des Zielblutdruckes bei Diabetes mellitus?

In epidemiologischen Studien fand sich ein enger Zusammenhang zwischen dem Auftreten von makrovaskulären Komplikationen wie Schlaganfall, koronarer Herzkrankheit (KHK), peripherer arterieller Verschlusskrankheit (PAVK) sowie mikrovaskulären Komplikationen (diabetische Nierenerkrankung und diabetische Retinopathie) und den erhobenen Blutdruckwerten [10]. Da in epidemiologischen Analysen das niedrigste Risiko bei Blutdruckwerten von 120/80 mm Hg beobachtet wurde [13, 14], glaubte man, dass möglichst niedrige Blutdruckwerte mittels antihypertensiver Therapiemaßnahmen zu erzielen wären. Die Ergebnisse aus Beobachtungsstudien dürfen aber nicht unreflektiert in klinische Empfehlungen zur Intervention übersetzt werden, für den Nachweis der Effizienz und Sicherheit von Interventionen sind randomisiert kontrollierte Studien notwendig.

Während neuere Studienergebnisse randomisiert kontrollierter Studien ein niedriges Blutdruckziel von 120–129/70–79 mm Hg bei guter Verträglichkeit der Therapie bei Diabetes nahelegen, zeigen sie auch, dass der Zielblutdruck ähnlich wie bei der Intensität der Glukoseeinstellung [15] individualisiert werden muss.

In der BROAD-Studie [16] wurden 12.821 Menschen mit Typ-2-Diabetes und erhöhtem kardiovaskulärem Risiko randomisiert zu einem Zielblutdruck von < 120 mm Hg oder einer Standardbehandlung mit Zielblutdruck < 140 mm Hg systolisch. Der breite primäre Endpunkt von kardiovaskulärem Tod, nichttödlichem Schlaganfall, nichttödlichem Myokardinfarkt und Behandlung bzw. Hospitalisation wegen Herzinsuffizienz wurde in der intensiv behandelten Gruppe um 21 % reduziert, es wurden systolische Blutdruckwerte von 122 vs. 133 mm Hg in der intensiver therapierten vs. der weniger intensiv therapierten Gruppe erzielt. Unerwünschte Ereignisse insgesamt traten ähnlich häufig in den Behandlungsgruppen auf, allerdings kam es in der intensiver therapierten Gruppe häufiger zu symptomatischer Hypotonie und Hyperkaliämie.

Einen ähnlichen Ansatz verfolgte die ESPRIT-Studie [17]. Hier wurden 11.255 Menschen mit hohem kardiovaskulärem Risiko, von denen 39 % Typ-2-Diabetes hatten, einer intensiven Behandlung mit Zielblutdruck von systolisch < 120 mm Hg vs. einer Standardbehandlung mit Zielblutdruck < 140 mm Hg zugeordnet; die durchschnittlich erzielten systolischen Blutdruckwerte lagen bei 120 vs. 135 mm Hg. Der primäre Endpunkt (kardiovaskulärer Tod, Myokardinfarkt, Revaskularisierung, Hospitalisierung wegen Herzinsuffizienz, Schlaganfall) trat in der intensiver therapierten Gruppe signifikant weniger häufig auf, mit einer Reduktion des relativen Risikos von 12 %. Allerdings traten unerwünschte Ereignisse in der intensiver therapierten Gruppe etwas häufiger auf, im Besonderen war die Inzidenz von Synkopen signifikant erhöht (0,4 % vs. 0,1 %).

Im Blutdruckarm der ACCORD-Studie wurden Nachteile einer zu niedrigen Blutdruckeinstellung (< 120 mm Hg) nachgewiesen: Es wurden hier 4700 Menschen mit Typ-2-Diabetes entweder einer intensivierten Therapie (systolischer Zielblutdruck < 120 mm Hg) oder einem Standardtherapiearm (systolischer Zielblutdruck < 140 mm Hg) zugeordnet [18]. Nach ca. 5 Jahren betrugen die Blutdruckwerte 119 mm Hg vs. 133 mm Hg. Durch die intensivierte Therapiestrategie wurde der primäre Endpunkt schwerer kardiovaskulärer Ereignisse nicht signifikant gesenkt, auch die Mortalität wurde nicht beeinflusst; Schlaganfälle nahmen allerdings um 47 % ab. Die intensivierte Therapie war jedoch mit kardialen Arrhythmien, Hyperkaliämie und Hypotension assoziiert. Subsets der Menschen in ACCORD mit hohem kardiovaskulärem Risiko profitierten allerdings von einer aggressiven Blutdruckeinstellung [19, 20].

In einer observationellen Studie von Cooper-DeHoff et al. [21] fand sich bei Menschen mit Diabetes und koronarer Herzerkrankung unter einer intensivierten Blutdrucksenkung eine Zunahme der Gesamtmortalität; bei systolischen Blutdruckwerten < 110 mm Hg war die Mortalität im Vergleich zu systolischen Blutdruckwerten von 125–130 mm Hg signifikant erhöht. Auch in der VADT-Studie fand sich eine signifikante Zunahme der kardiovaskulären Ereignisse, wenn die diastolischen Blutdruckwerte < 70 mm Hg abgesenkt wurden [22].

## Blutdruckzielwerte verschiedener Fachgesellschaften

Die aktuellen Leitlinien der American Diabetes Association (ADA) 2026 [23] fordern ein generelles Blutdruckziel von < 130/90 mm Hg, wenn dieser Blutdruck sicher erreicht werden kann; ein niedrigeres Blutdruckziel < 120 mm Hg systolisch wird bei hohem kardiovaskulärem oder renalem Risiko empfohlen. Die Guidelines von American College of Cardiology/American Heart Association geben für Menschen mit Diabetes mellitus dieselbe Empfehlung [2]; Blutdruckzielwerte von < 130/80 mm Hg werden auch von kanadischen Experten [24], von japanischen Experten [25] sowie von der IDF [9] empfohlen. UK-NICE empfiehlt einen Zielblutdruck < 140/90 mm Hg bei einem Alter < 80 und von < 150/90 mm Hg in einem Alter von ≥ 80 Jahren [26].

Die 2024 publizierten Hypertonieleitlinien der ESC [1] empfehlen für Menschen mit Diabetes einen Zielbereich von 120–129/70–79 mm Hg, wenn die Behandlung gut vertragen wird; weniger strenge Blutdruckzielwerte von etwa < 140 mm Hg systolisch werden nur bei symptomatischer orthostatischer Hypotonie und bei einem Alter ≥ 85 Jahre formuliert. Eine medikamentöse antihypertensive Behandlung soll begonnen werden, wenn nach 3 Monaten Lebensstilintervention der beim Arzt gemessene Blutdruck ≥ 130/80 mm Hg liegt. Ältere ESC-Guidelines erlaubten auch höhere Zielwerte [27, 28].

## Warum ist das alles so kompliziert?

Die Studienlage hinsichtlich intensivierter Blutdrucksenkung ist komplex und wird teilweise widersprüchlich interpretiert, wobei dies insbesondere für die ACCORD-BP [18] gilt, in der die intensivere Blutdrucksenkung keinen überzeugenden Vorteil hinsichtlich der Prävention des primären Endpunktes zeigte, bei einer erhöhten Inzidenz von unerwünschten Ereignissen unter intensiverer Blutdrucktherapie. Demgegenüber zeigen die neueren Studien BROAD [16] und ESPRIT [17], wie oben ausgeführt, durchaus einen Vorteil durch intensivere Blutdrucksenkung.

Es ist unklar, ob die unterschiedlichen Studienergebnisse auf die differenten Populationen zurückzuführen sind oder ob auch das Studiendesign einen Einfluss hatte [29]. Auch die unterschiedliche Blutdruckmessung und die limitierte statistische Power (die 95 %-Konfidenzintervalle überlappen sich) müssen dabei bedacht werden.

In der ACCORD-Studie wurde gleichzeitig auch der Effekt einer intensivierten Blutzuckerkontrolle (HbA_1c_-Zielwert < 6,0 %) untersucht, wobei in der intensivierten Gruppe eine erhöhte kardiovaskuläre Mortalität beobachtet wurde [30]. In einer Re-Analyse der ACCORD-Studie [31] fand sich im glykämischen Standardarm unter intensivierter Blutdrucksenkung eine signifikante Senkung von Schlaganfällen (−39 %), Myokardinfarkten (−37 %) und CV-Endpunkten insgesamt (−33 %); Mancia und Grassi [6] diskutieren einen potenziell negativen Effekt von schweren Hypoglykämien im intensivierten Blutzuckerarm auf den potenziell vorteilhaften Effekt einer intensivierten Blutdruckeinstellung.

In einer gepoolten Analyse von ACCORD-BP und SPRINT fanden sich ähnlich günstige Effekte auf kardiovaskuläre Endpunkte unter einer intensivierten Blutdruckkontrolle mit fehlender statistischer Heterogenität [20]. In einer weiteren gepoolten Sekundäranalyse von ACCORD-BP und SPRINT fanden sich hingegen warnende Hinweise darauf, dass eine zu strikte Blutdruckeinstellung die Inzidenz der chronischen Niereninsuffizienz (CNI) steigern dürfte [32]: Bei Menschen ohne CNI vor Intervention (*n* = 4311 in ACCORD; *n* = 6715 in SPRINT) wurde der Effekt der systolischen Blutdrucksenkung (13,9 mm Hg in ACCORD und 15,2 mm Hg in SPRINT) auf einen Abfall der eGFR > 30 % auf < 60 ml/min per 1,73 m^2^ analysiert. Nach 3 Jahren betrug die kumulative Inzidenz einer CNI in der ACCORD-Studie 10 % im intensivierten Arm vs. 4,1 % im Standardarm; auch in der SPRINT-Studie war die Inzidenz einer CNI signifikant höher im intensivierten Arm: 3,5 % vs. 1,0 % im Standardarm. Eine Überwachung der Nierenfunktion unter intensivierter Blutdruckkontrolle ist daher sehr wichtig.

Bezüglich der Zielwerte für den diastolischen Blutdruck besteht weniger Diskrepanz der Expertenmeinung. In der HOT(Hypertension Optimal Treatment)-Studie fand sich bei Typ-2-Diabetes [33] mit diastolischen Blutdruckzielwerten < 80 mm Hg (erreichter Zielwert betrug 81 mm Hg) eine 51 %-Senkung der CV-Ereignisse im Vergleich zur Kontrollgruppe (< 85 mm Hg oder < 90 mm Hg). In der UKPDS-Studie [34] führte die Senkung des diastolischen Blutdruckes von 87 auf 82 mm Hg zu einer signifikanten Abnahme von Myokardinfarkt (−21 %) und Schlaganfall (−44 %). In der SAVOR-Studie [35] fand sich die niedrigste CV-Ereignisrate bei diastolischen Blutdruckwerten zwischen 80 und 90 mm Hg und systolischen Blutdruckwerten zwischen 130 und 140 mm Hg. Diastolische Blutdruckwerte < 60 mm Hg waren mit einem erhöhten Myokardinfarktrisiko assoziiert (HR 2,30 [95 % CI 1,50–3,53]) im Vergleich zu höheren diastolischen Blutdruckwerten (80–90 mm Hg). Die ADA Guidelines [3] empfehlen diastolische Blutdruckwerte < 80 mm Hg, die ESC Guidelines [1] empfehlen diastolische Blutdruckwerte von 70–79 mm Hg, sie geben eine Klasse-IIb-Empfehlung für die Intensivierung der antihypertensiven Therapie, wenn die systolischen, nicht aber die diastolischen Blutdruckwerte im Zielbereich sind – dies kann also erwogen werden, um das kardiovaskuläre Risiko weiter zu senken.

## Therapie mit Antihypertensiva

Bei bestätigtem erhöhtem Blutdruck (Blutdruck ≥ 130/80 mm Hg bei mehreren Messungen an verschiedenen Tagen) sollte bei den meisten Menschen mit Diabetes eine medikamentöse Blutdrucktherapie begonnen werden, wenn der Blutdruck nach 3 Monaten Lebensstilmodifikation nicht im Zielbereich ist. Zumindest bei unbehandelten Blutdruckwerten von ≥ 150/90 mm Hg sollte bereits initial eine Kombination von 2 antihypertensiven Wirkstoffen gegeben werden, wobei die ESC eine niedrig dosierte Kombinationstherapie bereits bei niedrigeren Blutdruckwerten empfiehlt. Es sollten Antihypertensiva verwendet werden, für die bei Diabetes eine Reduktion von kardiovaskulären Ereignissen gezeigt wurde: ACE-Hemmer, Angiotensin-2-Rezeptorblocker, Diuretika vom Thiazid-Typ und Kalziumantagonisten vom Dihydropyridin-Typ. Bei Albuminurie oder KHK wird ein ACE-Hemmer empfohlen; ACE-Hemmer bzw. Angiotensinrezeptorblocker wirken potent in der Kombination mit Diuretikum und Kalziumkanalblocker. Sie werden in der vorliegenden Leitlinie als antihypertensive Wirkstoffe der ersten Wahl empfohlen. Kontraindiziert sind ACE-Hemmer bzw. Angiotensinrezeptorblocker allerdings während einer Schwangerschaft.

Bei signifikanter Albuminurie (Albumin/Kreatinin-Ratio ≥ 30 mg/g) sollte ein ACE-Hemmer oder ein Angiotensinrezeptorblocker (ARB) in der höchsten für die Hypertoniebehandlung zugelassenen und verträglichen Dosierung gegeben werden. Dies gilt nicht nur bei Hypertonie: Unabhängig vom Blutdruck sollten ARBs oder ACE-Hemmer bei Albuminurie eingesetzt werden. Unter Behandlung mit einem ACE-Hemmer, einem Angiotensin-2-Rezeptorblocker oder einem Diuretikum sollte die Nierenfunktion zumindest 1‑mal im Jahr kontrolliert werden. Bei Diabetes mit Nierenerkrankung ist es besonders schwierig, die Blutdruckzielwerte zu erreichen, sodass bei der Mehrzahl der Betroffenen eine antihypertensive Kombinationstherapie erforderlich ist.

Die Kombination von ACE-Hemmern und Angiotensinrezeptorblockern ist zur Blutdrucksenkung nicht sinnvoll, eine Kombination dieser beiden Medikamentenklassen mit direkten Renininhibitoren ist kontraindiziert. Es wurde in der Vergangenheit versucht, durch eine Doppelblockade des Renin-Angiotensin-Aldosteron-Systems die Progression der Albuminurie besser zu beeinflussen als mit einer Monotherapie mittels ACE-Hemmer, ARB oder Renininhibitoren. Bei diesen Studien – ONTARGET [36, 37], ALTITUDE [38] – zeigte sich zwar eine stärkere Reduktion der Albuminurie durch die duale Blockade, schwere Nebenwirkungen (unter anderem Hypotonie und Hyperkaliämie) wurden allerdings unter dieser Kombinationstherapie signifikant häufiger beobachtet.

Lange Zeit wurden Kombinationstherapien von ACE-Inhibitoren oder ARB mit niedrig dosierten Diuretika favorisiert. In der ACCOMPLISH-Studie [39] wurde allerdings bei 6000 Menschen mit Typ-2-Diabetes und Hypertonie nachgewiesen, dass bei gleichen Blutdruckzielwerten (135/80 mm Hg) die Kombination von ACE-Inhibitoren mit Amlodipin der Kombination von ACE-Inhibitoren und Hydrochlorothiazid deutlich überlegen war. Die Überlegenheit könnte aber auch darauf zurückzuführen sein, dass die Halbwertszeit von Amlodipin wesentlich länger ist als jene von Hydrochlorothiazid. Da Menschen mit Diabetes häufig – insbesondere, wenn sie mit Insulin behandelt sind – eine vermehrte Wasserretention aufweisen, ist eine zusätzliche Therapie mit niedrig dosierten Diuretika im Sinne einer Dreifachkombination häufig sinnvoll.

In einer Blutdruckzielwertstudie für die Sekundärprävention an 29.000 Menschen mit Typ-2-Diabetes in Deutschland und Österreich [40] erreichten 67 % der Betroffenen nach Herzinfarkt den Blutdruckzielwert 130/80 mm Hg und 90 % nach Schlaganfall den Zielwertbereich 120/70–140/90. In einer kanadischen Studie erreichten nur 36 % der im allgemeinmedizinischen Bereich Betreuten einen Zielwert von < 130/80 m Hg [41].

Mancia und Grassi [6] berichteten, dass in prospektiven randomisierten Studien bei hypertensiven Menschen mit Diabetes nur in 5 von 13 Studien systolische Blutdruckzielwerte ≤ 140 und nur in 6 von 13 Studien diastolische Blutdruckzielwerte ≤ 80 mm Hg erreicht wurden.

Bei inadäquat kontrollierten Blutdruckwerten trotz einer Dreifachkombination antihypertensiver Wirkstoffe, die auch ein Diuretikum einschließt, soll die zusätzliche Gabe eines Mineralokortikoidantagonisten zur Blutdruckkontrolle erwogen werden. Unter dieser Kombinationstherapie sind allerdings regelmäßige Kontrollen der Nierenfunktion und des Kaliums unabdingbar. In der FIDELIO-Studie [42] brachte die zusätzliche Gabe von Finerenon zu ACE-Hemmer/Angiotensinrezeptorblocker bei Typ-2-Diabetes und chronischer Nierenerkrankung eine signifikante Reduktion renaler wie auch kardiovaskulärer Ereignisse.

## Blutdrucksenkung durch Antidiabetika

Bei Verwendung von mehreren Antidiabetikaklassen kommt es zu einer signifikanten Blutdrucksenkung, wodurch die Blutdruckeinstellung verbessert wird. Während unter Metformin, Insulin, Sulfonylharnstoffen und DPP-4-Inhibitoren keine Veränderung der Blutdruckwerte beobachtet wurde, senken SGLT-2-Inhibitoren, GLP-1-Rezeptoragonisten, Tirzepatid und Pioglitazon den Blutdruck signifikant.

In einer Metaanalyse von Qayyum [43] an 13 Studien senkten die Glitazone im Vergleich zu Placebo den systolischen/diastolischen Blutdruck signifikant um 4,9/1,8 mm Hg. In einer Metaanalyse von Wang et al. [44] wurde der blutdrucksenkende Effekt von GLP-1-Rezeptoragonisten in 13 randomisiert kontrollierten Studien (RCTs) analysiert. Exenatid senkte im Vergleich zu Placebo den systolischen/diastolischen Blutdruck um 5,2 und 5,4 mm Hg und Liraglutid senkte den systolischen Blutdruck um 5,6 mm Hg.

Eine rezentere Metaanalyse [45] fand im Vergleich zu Placebo Senkungen des systolischen Blutdrucks unter Semaglutid, Liraglutid, Duraglutid und Exenatid im Ausmaß von −1,5 bis −3,4 mm Hg, eine Senkung des diastolischen Blutdrucks wurde nur unter Exenatid beobachtet, um 0,9 mm Hg. Die Veränderungen des systolischen Blutdrucks waren assoziiert mit Veränderungen des Body-Mass-Index unter den GLP-1-Agonisten, was darauf hinweist, dass die Blutdrucksenkung unter GLP‑1 Agonisten primär durch die darunter erzielte Gewichtsreduktion bedingt ist.

Für den kombinierten GIP/GLP-Rezeptoragonisten Tirzepatid, welcher besonders potent das Körpergewicht reduziert, wurden in einer Metaanalyse von 7 randomisiert kontrollierten Studien signifikante Blutdrucksenkungen von etwa 5 mm Hg beschrieben [46].

In einer Metaanalyse von Baker et al. [47] an 6 Studien, in denen unter einer Therapie mit SGLT-2-Inhibitoren 24-Stunden-Blutdruckmessungen durchgeführt wurden, fanden sich signifikante Blutdrucksenkungen systolisch um 3,8 mm Hg und diastolisch um 1,8 mm Hg. Die Blutdrucksenkung während des Tages betrug 4,3 mm Hg und während der Nacht 2,6 mm Hg. Die Blutdrucksenkungen waren unter den 4 getesteten SGLT-2-Inhibitoren (Canagliflozin, Dapagliflozin, Empagliflozin und Ertugliflozin) nicht signifikant unterschiedlich und korrelierten auch nicht mit dem Ausgangsblutdruck oder der Gewichtsabnahme. Der blutdrucksenkende Effekt der SGLT-2-Inhibitoren ist bezüglich der Nephroprotektion und Kardioprotektion prinzipiell als günstig zu beurteilen.

In der EMPA-REG Outcome-Studie [48], in der CANVAS-Studie [49] und in DECLARE [50] lagen die Ausgangsblutdrucke bei 135/77 mm Hg, 136/78 mm Hg und 135/85 mm Hg; es wurden ca. 80–90 % der teilnehmenden Menschen mit einem oder mehreren Antihypertensiva behandelt. Erwartungsgemäß sank der Blutdruck in diesen Studien unter den SGLT-2-Inhibitoren signifikant weiter ab. In der EMPA-REG Outcome-Studie kam es unter Empagliflozin zu einer hochsignifikanten Senkung von kardiovaskulärem Tod, Gesamtmortalität und Progression der Nierenerkrankung, der Blutdruckabfall von 135/77 mm Hg auf 131/75 mm Hg hatte keinerlei negativen Effekt. Diese Daten weisen darauf hin, dass Blutdruckwerte um 130 mm Hg auch bei Hochrisikokonstellation mit Typ-2-Diabetes nicht gefährlich sein dürften. Bei älteren Menschen sollte bei einer Kombinationstherapie von Antihypertensiva und SGLT-2-Inhibitoren eine häufigere Blutdruckmessung erfolgen, um im Bedarfsfall die Zahl oder Dosis der Antihypertensiva zu reduzieren.

## Blutdruckziele bei Diabetes mellitus je nach Komorbiditäten

### Blutdruckzielwerte und antihypertensive Therapie bei alten Menschen mit Diabetes

Es existieren nur wenige Studien, die speziell ältere Menschen mit Diabetes mellitus eingeschlossen haben, obwohl 30–60 % aller Menschen mit Diabetes in der Welt älter als 65 Jahre sind [51]. Ältere Menschen stellen eine sehr heterogene Gruppe dar, nicht wenige Menschen zwischen 65 und 75 Jahren sind frei von Komplikationen, generell ist aber die Komorbidität in dieser Altersgruppe deutlich erhöht (vor allem Herzinsuffizienz und Nierenerkrankung). Die STEP-Studie [52] schloss 8511 chinesische Menschen im Alter von 60 bis 80 Jahren ein, die zu einem systolischen Zielblutdruck von 110–130 vs. zu einem systolischen Zielblutdruck von 130–150 mm Hg randomisiert wurden. Die erreichten mittleren Blutdruckwerte lagen bei 128 vs. 136 mm Hg. Der recht breit definierte primäre Endpunkt kardiovaskulärer Ereignisse wurde mit der stärkeren Blutdrucksenkung um 24 % signifikant reduziert. Knapp 20 % der an dieser Studie Teilnehmenden hatten Diabetes; Subgruppenanalysen nach Diabetes-Status liegen nicht vor. Die 2024 publizierten Hypertonieleitlinien der ESC [53] empfehlen auch für ältere Menschen einen Blutdruckzielbereich von 120–129/70–79 mm Hg, wenn die Behandlung gut vertragen wird; weniger strenge Blutdruckzielwerte von etwa < 140 mm Hg systolisch werden allerdings in einem Alter ≥ 85 Jahre empfohlen. Auch die Empfehlungen der amerikanischen Diabetesgesellschaft fordern für die meisten älteren Menschen mit Diabetes einen Blutdruck von < 130/80 mm Hg, wenn dieser sicher erreicht werden kann. Ein weniger strenges Blutdruckziel von etwa < 140/90 mm Hg wird für ältere Menschen mit schlechtem Gesundheitszustand, limitierter Lebenserwartung oder hohem Risiko für unerwünschte Nebenwirkungen der antihypertensiven Therapie empfohlen.

### Nierenerkrankung

Eine internationale Expertengruppe hat für KDIGO (Kidney Disease Improving Global Outcomes) evidenzbasierte Richtlinien für die antihypertensive Behandlung bei Diabetes mit chronischer Nierenerkrankung festgelegt. Es wird hier bei chronischer Nierenerkrankung ein strenger Zielwert für den systolischen Blutdruck von < 120 mm Hg, sofern toleriert, gefordert [54], wobei diese Empfehlung kontrovers diskutiert wird [55].

Verschiedene Studien haben gezeigt, dass unterschiedliche Blutdruckzielwerte unterschiedliche Effekte auf Endorganschäden ausüben [18, 56]. Während möglichst niedrige Blutdruckwerte für die Schlaganfallprävention [18] und zur Verhinderung der Progression der diabetischen Nierenerkrankung [56] sehr günstig sein dürften, werden kardiovaskuläre Ereignisse insgesamt oder auch die Sterblichkeit dadurch nicht gesenkt. Unter besonders niedrigen Blutdruckwerten (sowohl systolisch als auch diastolisch) wurden sogar signifikant mehr kardiovaskuläre Ereignisse beobachtet, In der ROADMAP-Studie [57] wurde etwa mittels einer aggressiven Blutdrucksenkung (Zielblutdruck < 130/80 mm Hg) das Auftreten einer Mikroalbuminurie zwar signifikant verzögert, gleichzeitig stiegen aber die fatalen kardiovaskulären Ereignisse signifikant an, wobei dies primär auf vermehrte kardiovaskuläre Todesfälle bei präexistenter KHK zurückzuführen war.

In einer Metaanalyse von 24 RCTs an nahezu 70.000 Teilnehmenden (14 Studien mit Menschen mit Diabetes und 10 Studien mit Menschen ohne Diabetes) wurde unter einer Blutdrucksenkung von 10/5 mm Hg eine hochsignifikante Reduktion der dialysepflichtigen Niereninsuffizienz (ESKD) um 21 % nachgewiesen mit einer Number-Needed-to-Treat (NNT) von nur 8 bei einer Therapiedauer von 5 Jahren [58] Bemerkenswert ist allerdings der hochsignifikante Unterschied (*p* < 0,0001) zwischen Menschen mit Diabetes (HR 0,79 [0,66–0,95]) und jenen ohne Diabetes (HR 1,01 [0,81–1,26]). Diese Metaanalyse würde also nahelegen, dass eine Blutdrucksenkung bei Menschen ohne Diabetes keine Senkung des Risikos einer dialysepflichtigen Niereninsuffizienz bewirkt, während bei Menschen mit Diabetes mellitus eine relativ geringe Blutdrucksenkung von 10/5 mm Hg eine dramatische Verbesserung bewirken könnte.

Allerdings waren sowohl in ACCORD-BP- als auch in der SPRINT-Studie [32] Teilnehmende im intensivierten Arm einem höheren Risiko für das Entstehen einer CNI (eGFR-Abfall > 30 % auf < 60 ml/min per 1,73 m^2^) ausgesetzt als im Standardarm. Eine Überwachung der Nierenfunktion unter intensivierter Blutdruckkontrolle erscheint daher sehr wichtig.

### Schlaganfall

Xie et al. [59] analysierten den Effekt einer Blutdrucksenkung auf das Schlaganfallrisiko in einer Network-Analyse aus 21 RCTs mit insgesamt 65.000 Teilnehmenden. Untersucht wurde die Risikoreduktion einer Blutdrucksenkung von 10/5 mm Hg bei systolischen Ausgangsblutdruckwerten > 140, < 140, > 130 und < 130 mm Hg. Für die niedrigsten Ausgangsblutdruckwerte (< 130 mm Hg) fand sich noch immer eine signifikante Schlaganfallreduktion bei Menschen mit Diabetes (HR 0,61 [95 % CI: 0,47–0,79]).

In der ACCORD-BP-Studie [18] wurde unter der intensivierten Blutdrucksenkung mit einem Zielwert von 119,5 mm Hg im Vergleich zum Kontrollarm (133,5 mm Hg) ebenfalls eine hochsignifikante Risikoreduktion des Schlaganfalls (HR 0,59 [95 % CI: 0,39–0,89]) beobachtet.

### KHK und PAVK

Eine Auswertung von Daten der SAVOR TIMI 53-Studie [35] zeigte das niedrigste kardiovaskuläre Risiko von Menschen mit KHK bei Blutdruckwerten von 130–140 mm Hg systolisch und von 80–90 mm Hg diastolisch. Diese Daten waren allerdings Beobachtungsdaten, nicht Ergebnisse einer randomisierten Intervention zur Beurteilung verschiedener Blutdruckziele. Die aktuellen ESC-Empfehlungen zur stabilen koronaren Herzkrankheit empfehlen deshalb dieselben Blutdruckziele bei KHK wie in anderen Hochrisikosituationen, also ein Blutdruckziel von 120–130/70–79 mm Hg [60].

Menschen mit Diabetes und PAVK haben eine weitaus schlechtere Prognose als Menschen mit Diabetes nach Herzinfarkt oder Schlaganfall, wie in der PROactive-Studie nachgewiesen werden konnte [61]; eine rezente österreichische Studie zeigt ähnliche Ergebnisse [62]. In der Post-hoc-Analyse der INVEST-Studie [63] wurde das besonders hohe Risiko bei PAVK (Diabetesanteil 41 %) bestätigt. Bei insgesamt 2699 Menschen mit PAVK (1106 Menschen mit Typ-2-Diabetes) zeigte sich nach einer Studiendauer von 2,6 Jahren ein enger Zusammenhang zwischen den Blutdruckwerten und dem primären Endpunkt (Gesamtmortalität, nichtfataler Herzinfarkt und nichtfataler Schlaganfall). Der beste Outcome (niedrigste HR für den primären Outcome) fand sich bei systolischen Blutdruckwerten zwischen 135 und 145 mm Hg und diastolischen Werten zwischen 60 und 90 mm Hg. Bei niedrigeren systolischen Blutdruckwerten stieg die HR bis auf 1,7 (systolischer Blutdruck um 110 mm Hg) an, bei Blutdruckwerten um 180 mm Hg lag die HR sogar bei 2,5. Insgesamt ist die Datenlage bei PAVK und Diabetes mellitus besonders schwierig, da es wenig Primärstudien gibt. Die aktuellen ESC-Empfehlungen zur PAVK fordern bei PAVK dieselben Blutdruckziele wie für andere Hochrisikosituationen, also ein Blutdruckziel von 120–130/70–79 mm Hg [64].

## Metaanalysen

Eine große Metaanalyse mit Daten der Blood Pressure Lowering Treatment Trialists’ Collaboration schloss Studien ein, welche blutdrucksenkende Wirkstoffe gegen Placebo oder andere blutdrucksenkende Wirkstoffe verglichen, ebenso wie Studien, die eine intensivere vs. eine weniger intensive Blutdrucksenkung verglichen. Insgesamt wurden 51 randomisiert kontrollierte Interventionsstudien in die große Metaanalyse aufgenommen, mit insgesamt 358.533 Teilnehmenden, von denen 103.125 einen bei der Baseline bekannten Typ-2-Diabetes hatten. Der mittlere Blutdruck bei der Baseline bei Menschen mit vs. jenen ohne Diabetes war 149/84 vs. 153/88 mm Hg. Über ein medianes Follow-up von 4,2 Jahren führte eine Blutdruckreduktion um 5 mm Hg systolisch in beiden Gruppen zu einer Reduktion schwerer kardiovaskulärer Ereignisse, wobei der Behandlungseffekt bei Menschen mit Diabetes signifikant weniger stark ausgeprägt war als bei Menschen ohne Diabetes. Aufgrund des höheren absoluten kardiovaskulären Risikos profitierten Menschen mit Diabetes allerdings im gleichen Ausmaß wie jene ohne Diabetes [65].

Eine rezente Metaanalyse verglich die Auswirkungen einer intensiveren Blutdruckbehandlung auf kardiovaskuläre Ereignisse bei Menschen mit Diabetes mit einer Standardbehandlung. Als intensivere Behandlung wurden Blutdruckziele unter 130 mm Hg systolisch und unter 80 mm Hg diastolisch definiert. Acht Studien kamen in die Auswertung mit insgesamt 16.634 Teilnehmenden. Die intensivere Blutdruckbehandlung reduzierte kardiovaskuläre Ereignisse insgesamt, am deutlichsten Schlaganfälle, nicht aber koronare Ereignisse und nicht die Gesamtsterblichkeit [66].

Aggarwal et al. [67] analysierten den Effekt von Blutdruckzielwerten < 120 mm Hg vs. < 140 mm Hg an 14.000 Menschen mit und ohne Diabetes aus den kontrollierten Studien von ACCORD-BP und SPRINT. Jene mit den niedrigen Blutdruckzielwerten hatten signifikant weniger CV-Ereignisse (HR 0,83; 95 % CI 0,74–0,92; *p* < 0,001), eine Interaktion mit Diabetes war nicht nachweisbar, wenngleich die positiven Effekte einer intensiven Blutdrucksenkung bei Diabetes viel stärker ausgeprägt waren. Thomopolous et al. [58] analysierten das Ausmaß einer Blutdrucksenkung von 10/5 mm Hg in 27 RCTs bei Menschen mit Diabetes und in 28 RCTs bei Menschen ohne Diabetes in Abhängigkeit vom Ausgangsblutdruck (> 140, 130–140 und < 130 systolisch). Während die Blutdrucksenkung auch bei niedrigen Ausgangsblutdruckwerten ohne Diabetes für alle Endpunkte positive Effekte zeigte, fand sich dies bei Diabetes nur für den Endpunkt Schlaganfall, ein signifikanter Anstieg von Gesamtmortalität und kardiovaskulärem Tod wurde allerdings nicht beobachtet. Aus einer weiteren Metaanalyse hat dieselbe Arbeitsgruppe den Schluss gezogen, dass Blutdruckwerte um 130 mm Hg mit dem geringsten vaskulären Risiko assoziiert sein dürften [68], eine ganz ähnliche Beurteilung kommt von der Arbeitsgruppe von Sarafidis [69]. Eine umfangreiche Analyse von Mancia und Grassi [6] schließt mit der Feststellung, dass die Blutdruckzielwerte bei Diabetes wahrscheinlich niedriger als < 140/90 sein sollten und wahrscheinlich 130/80 mm Hg betragen sollten. In einer weiteren Metaanalyse, die Daten von über 340.000 Studienteilnehmern verwendete, war, ohne dass hier das Vorliegen eines Diabetes berücksichtigt worden wäre, eine 5 mm Hg-Senkung des systolischen Blutdrucks mit einer Risikoreduktion schwerer kardiovaskulärer Ereignisse um 11 % (bei vorbestehender kardiovaskulärer Erkrankung) bzw. 9 % (ohne vorbestehende kardiovaskuläre Erkrankung) assoziiert – unabhängig vom systolischen Ausgangsblutdruck [70].

## Zusammenfassung

Internationale Fachgesellschaften empfehlen bei Diabetes Blutdruckzielwerte von in der Regel < 130/80 mm Hg, die ESC formuliert einen Zielbereich von 120–129/70–79 mm Hg.

Ähnlich wie bei der Blutzuckereinstellung erscheint es in Anbetracht der Evidenzlage aber sinnvoll, eine Individualisierung der Blutdruckzielwerte in Abhängigkeit von Alter und Komorbiditäten anzustreben. Besonders wichtig ist, dass zumindest ein Blutdruck < 140/90 mm Hg erzielt wird; dieses Ziel wird leider von vielen Betroffenen nicht erreicht. Menschen mit bestimmter Komorbidität – nach Schlaganfall oder bei Vorliegen einer Nierenerkrankung – profitieren besonderes von niedrigeren Zielwerten. Besonders niedrige Zielwerte (< 120/80 mm Hg) sind für die Primär- und Sekundärprävention von Schlaganfall und Nierenerkrankung prinzipiell günstig, wegen des erhöhten Auftretens von schwerwiegenden Nebenwirkungen ist hier allerdings Vorsicht angezeigt.

## Interessenkonflikt

J.M. Brix: Honorare für Advisory Board und Vorträge von Abbott, AstraZeneca, Bayer, Boehringer Ingelheim, Dexcom, Eli Lilly, Medtronic, Novo Nordisk. R. Klauser-Braun hat in Bezug auf diesen Artikel von folgenden Unternehmen, die teils auch fördernde Mitglieder der ÖDG sind, Forschungsunterstützungen und/oder Honorare erhalten: Amgen, AstraZeneca, Böhringer Ingelheim, Lilly, Menarini, Novartis, Novo, Ratiopharm, Sanofi-Aventis. E. Zitt hat in Bezug auf diesen Artikel von folgenden Unternehmen, die teils auch fördernde Mitglieder der ÖDG sind, Forschungsunterstützungen und/oder Honorare erhalten: AstraZeneca, Bayer Austria, Boehringer Ingelheim, CSL Vifor, GSK, Novartis, Otsuka Pharmaceutical, SOBI, STADA, Vantive. C.H. Saely, G.-H. Schernthaner, H. Drexel und G. Schernthaner geben an, dass kein Interessenkonflikt besteht.
